# 4277 Functional consequences of the juvenile idiopathic arthritis risk variant at 1q24.3

**DOI:** 10.1017/cts.2020.299

**Published:** 2020-07-29

**Authors:** Halima Moncrieffe, Katelyn Dunn, Yongbo Huang, Xiaoting Chen, Carl D. Langefeld, Matthew T. Weirauch, John B. Harley, Leah C. Kottyan, Susan D. Thompson, Juvenile Arthritis Consortium for the Immunochip

**Affiliations:** 1 Cincinnati Children’s Hospital; 2 Wake Forest School of Medicine

## Abstract

OBJECTIVES/GOALS: Juvenile idiopathic arthritis (JIA) is the most common childhood rheumatologic disease childhood and a cause of pain and potential disability. JIA has a strong genetic component and no known cure. The goal of this study is to evaluate allele-dependent effects of a novel JIA risk variant at 1q24.3. METHODS/STUDY POPULATION: JIA patients meeting criteria for the two most common disease subtypes (oligoarticular and RF neg polyarthritis) were genotyped using the Immunochip, an Illumina array with dense coverage of the HLA region and 186 other loci previously reported in autoimmune diseases. Phase I association findings (Hinks, 2013) and Phase II analysis (unpublished) of an expanded cohort (4,271 JIA and 14,390 controls) identified new risk loci, including rs78037977 at 1q24.3. We prioritized rs78037977 and predicted possible impacted mechanisms based on Bayesian predictions of attributable risk, the surrounding chromatin landscape, and transcription factor binding data. A luciferase reporter assay was used to assess allele-dependent enhancer activity. RESULTS/ANTICIPATED RESULTS: rs78037977 is located between *FASLG* and *TNFSF18* at chromosome 1q24.3 is associated with JIA (p = 6.3x10^−09^), and explains 94% of the posterior probability at this locus; no other SNPs in linkage disequilibrium (r^2^>0.6). The chromatin landscape around rs78037977 contains H3K4Me1 and H3K27Ac marks, which are indicative of enhancer activity. Further, >160 transcription factors have chromatin immunoprecipitation followed by sequencing (ChIP-seq) peaks overlapping rs78037977 in various cellular contexts. In luciferase reporter assays, the region around rs78037977 containing the reference A allele had ~2-fold increased enhancer activity compared to the non-reference allele. DISCUSSION/SIGNIFICANCE OF IMPACT: This work provides *in vitro* evidence to support allele-dependent enhancer activity of a novel JIA-risk variant at 1q24.3. Our ongoing work investigates the effect of the DNA-containing region of rs78037977 on gene expression and differential transcription factor binding at rs78037977.

